# Contribution of CT-Scan Analysis by Artificial Intelligence to the Clinical Care of TBI Patients

**DOI:** 10.3389/fneur.2021.666875

**Published:** 2021-06-10

**Authors:** Clément Brossard, Benjamin Lemasson, Arnaud Attyé, Jules-Arnaud de Busschère, Jean-François Payen, Emmanuel L. Barbier, Jules Grèze, Pierre Bouzat

**Affiliations:** Université Grenoble Alpes, Inserm, CHU Grenoble Alpes, U1216, Grenoble Institut Neurosciences, Grenoble, France

**Keywords:** traumatic brain injury, artificial intelligence, computed tomography, segmentation, classification, review

## Abstract

The gold standard to diagnose intracerebral lesions after traumatic brain injury (TBI) is computed tomography (CT) scan, and due to its accessibility and improved quality of images, the global burden of CT scan for TBI patients is increasing. The recent developments of automated determination of traumatic brain lesions and medical-decision process using artificial intelligence (AI) represent opportunities to help clinicians in screening more patients, identifying the nature and volume of lesions and estimating the patient outcome. This short review will summarize what is ongoing with the use of AI and CT scan for patients with TBI.

## 1. Introduction

Traumatic brain injury (TBI) is a leading cause of death and disability in young people. It affects more than one European in 400 (1). It therefore has a strong human and financial impact on society (2). If the initial aggression induces primary lesions that are instantaneous and unavoidable, it can also cause delayed lesions, named secondary injuries, which strongly influence the neurological outcome. Therefore, the critical care management of TBI patients is to limit secondary brain injuries with therapies that are adapted to patients' severity (3).

Assessment of severity of TBI relies on clinical examination and initial brain imaging. Clinical examination is poor at the early phase of TBI and is based on the pupillary reactivity and the Glasgow Coma Score (GCS) that classifies TBI in 3 stages: mild, moderate, and severe. As a result, GCS categories cover a large pattern of TBI and are unable to differentiate specific evolutions such as diffuse injuries or focal lesions (4). Brain imaging, mainly computed tomography (CT) scan (5), is an additional tool to better classify TBI patients. Based on the radiological reading findings, researchers have built imaging scores that describe and quantify brain lesions. Several scores have been developed, i.e., the Marshall classification (6), the Rotterdam score (7), the Stockholm score (8), and the Helsinki score (9). All scores are correlated with the neurological outcome defined as the Glasgow Outcome Scale (GOS) (9–12).

However, there are some drawbacks with the use of these CT scan scores. One point is their dichotomized answers, i.e., yes or no, that may underestimate the global effect of small intracerebral lesions. Despite their relative simplicity, inter-observer variability should be considered with the use of these scores. Chun et al. (13) measured this inter-observer agreement and found an average Cohen's Kappa coefficient of 0.57 for the detection of CT abnormalities, far from 1, corresponding to a full inter-observer agreement. Finally, categorizing each patient within a CT scan score is demanding in human resources that may explain why these scores are not used in clinical practice. In this context, the development of automated approaches to determine the nature and volume of traumatic brain injuries from CT scans has gained interest among clinicians.

**Figure 1:**
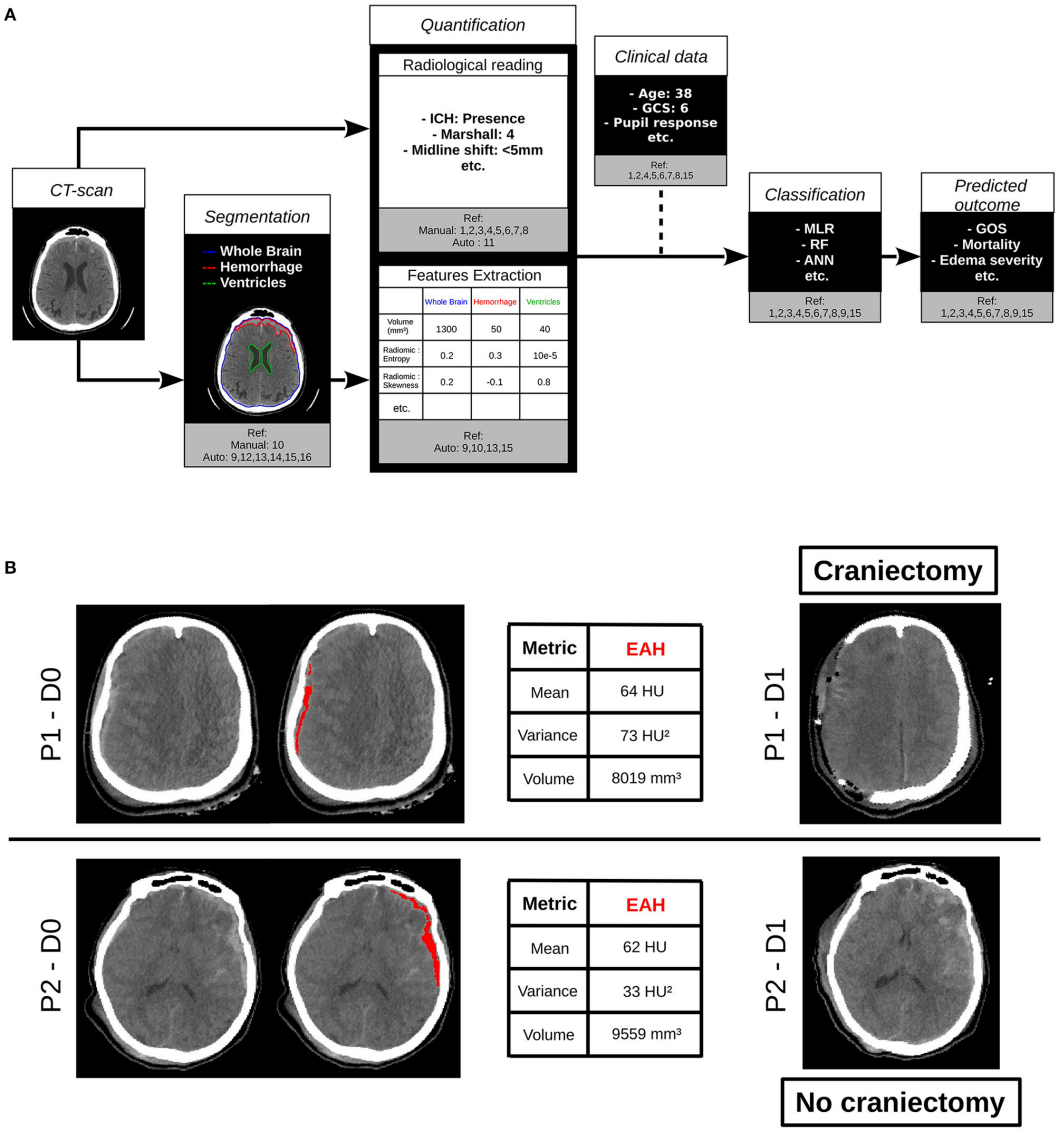
**(A)** Contribution of computed tomography (CT) scan analysis by artificial intelligence to the clinical care of traumatic brain injury (TBI) patients. References and terms are defined in Table 1. **(B)** Example of the use of artificial intelligence (AI) algorithms on clinical routine. CT scans of two patients (P1 and P2) at D0 were quantified with state of the art algorithms. On the right, CT scans of the same two patients acquired at D1 are shown. P1 and P2 had different clinical care. P1 underwent a decompressive craniectomy and not P2. Biggest extra axial hemorrhage (EAH) lesion was segmented with Brain Lesion Analysis and Segmentation Tool for Computed Tomography (BLAST-CT) (16) and radiomic metrics on this region of interest (ROI) were extracted as in (17). At first sight, the two lesions have the same profile, with equivalent volumes and means, but the variance of P1 is higher than twice the one of P2. That could for instance be a biomarker evaluated in further studies to predict the need for craniectomy. ICH, intracranial hemorrhage; GCS, Glasgow Coma Score; MLR, multivariate logical regression; RF, random forest; ANN, artificial neural network; GOS, Glasgow Outcome Score; CT scan, computed tomography image; Ref, References; HU, Hounsfield Units; ROI, region of interest; EAH, extra axial hemorrhage; D, day.

Due to the philosophical implications of intelligence's definition, the general definition of artificial intelligence (AI) remains unclear. Nevertheless, one can use the expression “AI” as defined in (14): *a field of computer science dedicated to the creation of systems performing tasks that usually require human intelligence*, and the term “machine learning” (ML) as: *a sub-field of AI that includes all those approaches that allow computers to learn from data without being explicitly programmed*. The rise of computing power capacities at the beginning of the 2,000 and the creation of large database have revealed the effectiveness of AI algorithms applied to medical images to improve the clinical care (14). Among the three ways of learning for ML algorithms (supervised, unsupervised, and reinforcement learning), the main algorithms used on biomedical imaging to predict outcome is the supervised learning, where input data and the variable to predict are known and the algorithms learn the relation between them (15). This expanding field of research may help the quantitative analysis of CT images and offer new perspectives in clinical care of TBI patients by following the process presented in Figure 1A. This review presents studies focusing on the classification and the segmentation of lesions based on the manual or automated analysis of CT scans. The main studies are summarized in Table 1. We will also cover the current research gaps and potential perspectives of the use of AI combined to CT scans in the TBI area.

**Table 1:** Summary of the main article cited in this review and their main properties.

**Reference**	**Task^a^**	**Input Data^b^**	**Output Data^c^**	**Nb subjects**	**Data selection^d^**	**Algorithm type^e^**	**Validation^f^**	**Evaluation metric^g^**	**Performance**	**Model available**
1) MRC CRASH Trial Collaborators (18)	Cla	Clinical data + RR	dGOS	18517	GCS ≤ 14	MLR	External	AUC	77%	Yes
2) Steyerberg et al. (19)	Cla	Clinical data + RR	dGOS	14781	GCS ≤ 12	MLR	External	AUC	80%	Yes
3) Raj et al. (9)	Cla	RR	dGOS	869	Severe + moderate + mild complicated TBI	MLR	Internal	AUC	75%	No
4) Matsuo et al. (20)	Cla	Clinical data + RR	dGOS	232	Abnormal RR	RF	Internal	AUC	89.5%	No
5) Hale et al. (21)	Cla	Clinical data + RR	dGOS	565	Mild + severe pediatric TBI	ANN	Internal	AUC	94.6%	No
6) Rau et al. (22)	Cla	Clinical data + RR	Mortality	2059	AIS≥3	MLR	Internal	Acc	93.5%	No
7) van der Ploeg et al. (23)	Cla	Clinical data + RR	Mortality	11026	Moderate + severe TBI	MLR	External	AUC	76.4%	No
8) Gravesteijn et al. (24)	Cla	Clinical data + RR	Mortality	12576	Moderate + severe TBI	GBM	External	AUC	83%	No
8) Gravesteijn et al. (24)	Cla	Clinical data + RR	dGOS	12576	Moderate + severe TBI	ANN	External	AUC	78%	No
9) Kim et al. (25)	Cla	CT-scan	Severe/mild edema	70	Pediatric TBI	Proportion of voxels ∈[17, 24] HU + non parametric tests	NI	AUC	85%	No
9) Kim et al. (25)	Cla	CT-scan	Delayed/mild edema	70	Pediatric TBI	Proportion of voxels ∈[17, 24] HU + non parametric tests	NI	AUC	75%	No
10) Rosa et al. (17)	Cla	CT-scan + lesions segmentation	EDH + SDH + Contusions	155	Presence lesion	Radiomic features extraction + PLS-DA	Internal	Acc	89.7%	No
11) Chilamkurthy et al. (26)	Cla	CT-scan	ICH + fracture + midline shift + mass effect	313809	NI	CNN	External	AUC	92.16 - 97.31%	No
12) Jadon et al. (27)	Seg	2D CT-scan	Hemmorhage	40000	NI	CNN	NI	DSC	85.78 - 94.24%	No
13) Jain et al. (28)	Seg	CT-scan	IC lesions	144	Center-TBI	CNN	Internal	DSC	73%	No
14) Kuo et al. (29)	Seg	CT-scan	ICH	791	NI	CNN	External	DSC	76.6%	No
15) Yao et al. (30)	Seg	CT-scan	Hematoma	828	GCS∈[4, 12]	CNN	Internal	DSC	69.7%	No
15) Yao et al. (30)	Cla	Clinical data + CT-scan	Mortality	828	GCS∈[4, 12]	RF	Internal	AUC	85.3%	No
16) Monteiro et al. (16)	Seg	CT-scan	IPH + EAH + PO + IVH	839	Center-TBI	CNN	Internal	DSC	36%	Yes
16) Monteiro et al. (16)	Cla	CT-scan	IPH + EAH + PO + IVH	490	Center-TBI + CQ500	CNN	External	AUC	83% - 95%	Yes

a *Task: Cla, Classification; Seg, Segmentation*.

b *Input Data: clinical data = metrics representing demography or physiology, RR, radiological reading metrics manually retrieved from CT scan and CT scan, computed tomography image*.

c *Output Data: dGOS, dichotomized Glasgow Outcome Score, EDH, extra dural hemmorhage; SDH, subdural hemorrhage; ICH, intracranial hemorrhage; IC, intracranial; PO, oerilesional edema; IVH, intraventricular hemorrhage*.

d *Data selection: GCS, Glasgow Coma Score; AIS, Abbreviated Injury Scale; NI, no information; Center-TBI and CQ500: public databases containing TBI CT scans*.

e *Algorithm type: MLR, multivariate logical regression; RF, random forest; ANN, artificial neural network; CNN, convolutional neural network; GBM, gradient boosting machine; HU, Hounsfield Units*.

f *Validation: NI, no information*.

g *Evaluation metric: AUC, area under the curve; Acc, accuracy; DSC, Dice similarity coefficient*.

## 2. Classification

### 2.1. Definition and Evaluation

Classification is the way for an algorithm to attribute a class to each input object, which is defined by one or more data points. There are different metrics to evaluate the performances of a classification. Let us assume a classification algorithm aimed at predicting a pathology and let us consider its evaluation on a test database. One defines as *TP* the number of true positive predictions, *TN* the number of true negative predictions, *FP* the number of false positive predictions, and *FN* the number of false negative predictions. The algorithm's Accuracy (*Acc*), which represents the percentage of good predictions, can be computed as:

$$\begin{array}{l} Acc=\frac{TP+TN}{TP+TN+FP+FN} \end{array}$$

To separate the cases of false prediction on healthy or pathological subjects, one can define the Sensitivity (*Se*) and Specificity (*Sp*):

$$\begin{array}{l} Se=\frac{TP}{TP+FN}\;Sp=\frac{TN}{TN+FP} \end{array}$$

Modifying the discrimination threshold of a classification algorithm allows to weigh the cost of predicting the pathology. It is indeed more costly to predict normality for a patient than pathology for a healthy subject. It allows to decrease *FN*, but as a consequence, increase *FP*, and modify *TP* and *TN*. The modification of this threshold leads to new values of *TP*, *TN*, *FP*, and *FN*, and one can summarize these values by representing *Se* in function of (1−*Sp*), defining the receiver-operator curve (ROC). The measure of the area under the curve (AUC) of the ROC curve represents the aggregate performances of the classification algorithm, whatever its discrimination threshold, and is a metric widely used to evaluate classification algorithms. An AUC of 1 describes a perfect classifier, and a value of 0.5 means that the classifier performs no better than chance.

### 2.2. State of the Art

#### 2.2.1. Manual Quantification

The first studies involving AI tools aimed to predict the outcome after TBI. Patient outcome at 6 and 12 months was defined favorable vs. unfavorable outcome, i.e., death and severe disability, according to the GOS score. From a methodological point of view, these studies were based on the analysis of heterogeneous metrics, representing demography (age, sex, etc.), physiology (pupil response to light, GCS score, blood glucose concentration, etc.), and brain CT scan metrics manually quantified by radiologists using imaging scores. These metrics were aggregated to build prognostic models using multivariate logical regression approaches as reviewed by Perel et al. (31). Two large datasets of TBI patients produced predictive models: CRASH (18) and IMPACT (19, 32, 33). These models were subsequently externally validated with a good performance in predicting mortality and unfavorable outcome (AUC between 0.65 and 0.87) (34). The prevalence of the metrics was estimated, and if the main predictors are age or GCS (35), CT characteristics manually quantified also carry crucial information able to predict TBI outcome (18, 36, 37). CT characteristics were also evaluated individually, and showed important prognostic ability in TBI outcome, as reviewed by Zhu et al. (38), but imaging scores were only able to predict outcome with an AUC between 0.56 and 0.82 (9, 11, 12).

More recently, instead of multivariate logical regression (MLR), complex algorithms were evaluated. In 2018, 9 ML algorithms were tested upon 232 TBI patients in order to predict outcome and mortality from 14 predictors including radiological reading findings at admission and at the following day (20). The best models were obtained using Random Forest (RF), providing an AUC of 0.895 for outcome prediction and using ridge regression, providing an AUC of 0.875 for mortality prediction, both on a dedicated test set.

Some other benchmarks of ML algorithms included artificial neural network (ANN) experiments (39). ANN-based models showed contrasted performances in classifying the outcome from manual quantified metrics, with studies obtaining results outperforming the state-of-the-art models, showing an AUC = 0.946 predicting the dichotomized GOS (21) or an Acc = 0.92 predicting 6-month mortality (22), and studies showing lower performances predicting in hospital mortality (AUC = 0.706) (23) or dichotomized outcome (AUC = 0.78) (24). These differences can be explained by the gap between the number of subjects of the study (all datasets included) (565, 2059, 11,026, 12,576, respectively, to the order of citation), the proportion of pathological and healthy subjects (6, 10, 26, and 47%) or by the validation method (internal, internal, external, external). These differences indicate that the methodology is a crucial step to truly measure ML model performances and to trust its predictions (40).

#### 2.2.2. Automated Quantification

Besides clinical metrics, CT scan provides high spatial resolution images of the brain that contain much more information than that summarized in imaging scores. Several researchers recently started to use automatic algorithms to exploit this large amount of CT scans with the aim to find biomarkers able to predict outcome in TBI.

##### 2.2.2.1. Histograms

To our knowledge, the first article to study TBI on CT scan was Kim et al. (25). Thanks to an automatic preprocessing pipeline of brain extraction of head injured pediatric patients, the authors showed that the proportion of brain voxel values ranging between 17 and 24 Hounsfield Units (HU) was a good predictor of edema severity (AUC = 0.85). This work demonstrated that (i) CT scans contain valuable quantitative information linked to the evolution of the status of the brain and (ii) automatic tools may be used to extract these quantitative features.

##### 2.2.2.2. Radiomics

Distribution of voxels values, represented by a histogram, is a simple metric to quantitatively characterize an image. One can go further and extract more complex metrics to represent shape, texture, or contrast of an image (41). This research field is called Radiomic (42), and has proven its relevance in tumor diagnosis (43), while its biological meaning is currently discussed (44). Each radiomic metric can be calculated on a 2D or 3D region of interest (ROI), leading to extract a large number of metrics characterizing the ROI. This has been employed in TBI patients (17), suggesting that extracting metrics of first-order statistics (FOS), texture, and shape from ROI of segmented lesions could discriminate the injury's nature, among epidural hematoma (EDH), acute subdural hematoma (ASH), and contusion, and providing an Acc = 89.7. Another way to use radiomic is to extract each metric from a sliding window moving on a CT scan, leading to generate as many parametric maps as metrics used. This method has been used by Muschelli et al. (45) to segment hemorrhage. However, this has not been specifically used for TBI yet to our knowledge.

##### 2.2.2.3. Convolutional Neural Networks

Deep learning is a subset of ML methods based on ANN. There are several types of networks among which recurrent neural networks (RNN) or convolutional neural networks (CNN). In computer vision, due to their ability to analyze pixels and their neighborhood, CNN are the most used networks in classification and segmentation tasks, and so in the biomedical imaging field (46, 47). In TBI, to classify injuries from CT scans, Chilamkurthy et al. (26) built several DL (and RF) algorithms each able to detect the presence or the absence of one type of lesion in a CT scan. Injury types contained different types of intracranial hemorrhage, fracture, midline shift, and mass effect. These algorithms were externally validated with an AUC from 0.92 to 0.97.

## 3. Automated Segmentation

### 3.1. Definition and Evaluation

Segmentation is, in image processing, an operation aimed to split an image in 2 or more ROI. Let us assuming a segmentation algorithm aimed to segment a brain lesion and its evaluation on a test database. To compare the output segmentation *X* to a ground truth segmentation *Y*, one can computes the Dice similarity coefficient (*DSC*), defined as, for |*X*| the number of pixels in the segmentation X:

$$\begin{array}{l} DSC=2*\frac{\left|X\cap Y\right|}{\left|X|+|Y\right|} \end{array}$$

This coefficient measures the number of common pixels between 2 segmentations in relation to the global number of pixels of the 2 segmentations. Some algorithms are trained on images that include images without lesion, therefore without ground truth segmentation. In these cases, if the algorithm predicts a lesion, even very small, the DSC is equal to 0 and strongly affects the mean DSC, although the segmentation error is quite small. That is why on some studies, DSC is only computed on large lesions, leading to better DSC values than studies measuring DSC on all lesions.

### 3.2. State of the Art

In computer vision segmentation, as well as in classification (see section 2.2.2.3), CNN is the most used ANN subtype.

#### 3.2.1. Segmentation

In TBI data, Jadon et al. (27) compared CNN architectures and built 3 algorithms, each segmenting one type of injury among (intraparenchymal hemorrhage [IPH], extra axial hemorrhage [EAH], and hemorrhagic contusions) from 2D CT slice. DSC ranged from 0.85 for EAH to 0.95 for IPH. On their side, Jain et al. (28) built an algorithm based on the segmentation of WM, GM, and CSF to segment acute intracranial lesions (DSC = 0.73) and cisterns (DSC = 0.70 for cisterns larger than 5 mL).

Kuo et al. (29) demonstrated the superiority of a training on random patches of 240*240 pixels and evaluation on sliding windows on a CT scan to detect and segment intracranial hemorrhage against more classical CNN, which takes as input a whole CT 2D image. The resulting algorithm provides an AUC of 0.966 on external validation and a DSC of 0.72 on internal validation.

As there are different ways to build a CNN, Yao et al. (30) built a multiview CNN aimed to segment acute hematoma that analyzes CT scan at different resolutions to help detecting small lesions. It shows a DSC of 0.69 on an internal test set containing 20 subjects.

More recently, Monteiro et al. (16) made available a model named Brain Lesion Analysis and Segmentation Tool for Computed Tomography (BLAST-CT), aimed to segment and classify four types of TBI injuries: IPH, EAH, intraventricular hemorrhage (IVH), and perilesional edema (PO). They built a CNN, inspired from the architecture of Deepmedic (48), and trained it on 839 TBI scans where the four former lesion types had been delineated by trained personnel. The resulting model's segmentation have been validated on an internal cohort of 655 scans, providing a DSC of 0.36 (as mentioned before, DSC is very sensitive to small volumes and might not be the best metric to evaluate segmentation of small lesions), while the classification has been validated on an external cohort of 490 scans, providing an AUC between 0.83 and 0.95 for the different lesion types to classify.

#### 3.2.2. Quantification From Segmentation

Besides this type of segmentation, some researchers went further in the quantification of CT images. Jain et al. (28) explored the measurement of cistern volume and midline shift because of a pipeline composed of CNNs and morphological operations and provides an Acc of 0.89 for midline shift estimation. Yao et al. (30) went, to our knowledge, the furthest. They automatically measured hematoma volumes, their localization, shape features representing the hematomas, and trained a RF algorithm with these metrics and biological information. Thereby, they were able to predict 6 months mortality, achieving an AUC of 0.85 on internal validation on 828 patients.

## 4. Discussions and conclusion

The development of ML algorithms offers new possibilities in predicting TBI outcome. While these methods provide few improvements in predicting outcome from clinical variables and imaging scores compared to classic multivariate logistic regression, they might change the paradigm about image quantification. In addition to the automatization and speeding up of the quantification, image processing methods can help to detect humanly undetectable patterns and, by represent early cerebral rearrangement, provide new biomarkers of TBI evolution, especially in TBI's modality of choice: CT.

### 4.1. Current Research Gaps

In the coming years, human segmentation and quantification of CT scans can probably be replaced by automated processing of ML algorithms, but their incorporation in clinical routine will be dependent on models and prediction's explainability and interpretability. Progress must be done on mastering CNN's theory to evaluate their capacities and limitations. A preliminary step could be the development of saliency maps (49), representing the voxels which have a strong impact on the prediction. Each prediction from a CT scan could be presented along with its saliency map, in order to explain the algorithm decision and potentially check its relevance, as already proposed by Kuo et al. (29).

Machine learning algorithms depend on their training dataset's nature. In order to deploy tools usable on multiple sites, one should put efforts on reducing intermachine and interprotocol variability. Indeed, the different models of scanner and parameters of acquisition, such as reconstruction algorithms, output resolution, voltage, or number of detectors, influence output images properties (50, 51). While researchers studied harmonization of metrics extracted from images (52), few studies intend to reduce variability at image level (53, 54). Since deep learning algorithms might be able to learn intersite variabilities and take them into account if trained on heterogeneous cohorts, efforts must then be made on the establishment of large and multicentric cohorts to cover the spectrum of all secondary injuries induced by TBI, as initiated by Maas et al. (55) or Flanders et al. (56).

As ML models on medical imaging usually follow the same pattern—*metric extractions, features selection, training, validation* and the same evaluation metrics (AUC, Acc, DSC, etc.)—it seems easy to compare them. However, these metrics strongly depend on the methodology of the model training and evaluation. If each study has its specifics, one must pay attention to the quality of the dataset (number of patients, heterogeneity/homogeneity), training and validation modalities (internal, external), and metrics of evaluation (DSC computed on all lesions or only on large lesions). An improvement in methodology's robustness, as well as in the availability of the mathematical models, will surely benefit all the scientific community and help build stronger studies, which are able to give birth to potential breakthrough in the clinical care of TBI patients.

### 4.2. Potential Future Developments

Brain CT represents the brain's density at the acquisition time. CT scans are generally acquired at different times to follow cerebral rearrangement after injuries or surgery. To improve our comprehension of cerebral rearrangement following heterogeneous secondary injuries, the establishment of longitudinal cohorts and their quantification is crucial to define different profile of evolution (5). Such profiles could then be isolated, leading to specific mechanisms and maybe specific treatments.

From a clinician point of view, the idea to combine clinical data and CT metrics is strongly relevant. One can imagine combining many variables from different nature, like most of the models presented in section 2.2.1, but replace manual quantification by automated and deep quantification of CT images provided by modern ML algorithms. As evaluated by Yao et al. (30), we also believe that in the field of TBI, localization of lesions have a large impact on the patient outcome. The development methods to register structural atlases on distorted TBI CT scans and the incorporation of this spatial information on prediction models might strongly improve their performances.

For research purposes, one might be soon able to predict clinical data as the neurological outcome or the level of medical care needed by a patient, as illustrated in Figure 1B. However, since no study evaluated the relevance of AI algorithms in the quantification of complex images, as mixed type injuries, artifacts altered images, or cohabitation of different lesions types as tumors or white matter diseases, their use in complex case studies remains challenging. One can then imagine that these algorithms will first be used in clinical routine for screening, triage, or indicative prediction in support of human readers.

### 4.3. Conclusion

AI algorithms have shown promising results in the biomedical field, especially in medical imaging. Their use on TBI CT scans, for classification and/or segmentation, is expanding and might become the reference methods in the next years if some problematic but surmountable issues are addressed. Their integration in clinical routine depends on the confidence on their predictions that might be increased with rigorous methodology.

## Author Contributions

CB, BL, and PB wrote the first draft of the manuscript. All authors listed have made a substantial, direct and intellectual contribution to the work, approved it for publication, proofread, and corrected the final manuscript.

## Conflict of Interest

The authors declare that the research was conducted in the absence of any commercial or financial relationships that could be construed as a potential conflict of interest.
